# Acquisition time reduction in pediatric ^99m^Tc‐DMSA planar imaging using deep learning

**DOI:** 10.1002/acm2.13978

**Published:** 2023-04-05

**Authors:** Shota Ichikawa, Hiroyuki Sugimori, Koki Ichijiri, Takaaki Yoshimura, Akio Nagaki

**Affiliations:** ^1^ Graduate School of Health Sciences Hokkaido University Sapporo Japan; ^2^ Department of Radiological Technology Kurashiki Central Hospital Kurashiki Okayama Japan; ^3^ Faculty of Health Sciences Hokkaido University Sapporo Japan

**Keywords:** acquisition time reduction, deep learning, image quality assessment, pediatric ^99m^Tc‐DMSA scintigraphy, renal uptake

## Abstract

**Purpose:**

Given the potential risk of motion artifacts, acquisition time reduction is desirable in pediatric ^99m^Tc‐dimercaptosuccinic acid (DMSA) scintigraphy. The aim of this study was to evaluate the performance of predicted full‐acquisition‐time images from short‐acquisition‐time pediatric ^99m^Tc‐DMSA planar images with only 1/5th acquisition time using deep learning in terms of image quality and quantitative renal uptake measurement accuracy.

**Methods:**

One hundred and fifty‐five cases that underwent pediatric ^99m^Tc‐DMSA planar imaging as dynamic data for 10 min were retrospectively collected for the development of three deep learning models (DnCNN, Win5RB, and ResUnet), and the generation of full‐time images from short‐time images. We used the normalized mean squared error (NMSE), peak signal‐to‐noise ratio (PSNR), and structural similarity index metrics (SSIM) to evaluate the accuracy of the predicted full‐time images. In addition, the renal uptake of ^99m^Tc‐DMSA was calculated, and the difference in renal uptake from the reference full‐time images was assessed using scatter plots with Pearson correlation and Bland–Altman plots.

**Results:**

The predicted full‐time images from the deep learning models showed a significant improvement in image quality compared to the short‐time images with respect to the reference full‐time images. In particular, the predicted full‐time images obtained by ResUnet showed the lowest NMSE (0.4 [0.4−0.5] %) and the highest PSNR (55.4 [54.7−56.1] dB) and SSIM (0.997 [0.995−0.997]). For renal uptake, an extremely high correlation was achieved in all short‐time and three predicted full‐time images (*R* ^2^ > 0.999 for all). The Bland–Altman plots showed the lowest bias (−0.10) of renal uptake in ResUnet, while short‐time images showed the lowest variance (95% confidence interval: −0.14, 0.45) of renal uptake.

**Conclusions:**

Our proposed method is capable of producing images that are comparable to the original full‐acquisition‐time images, allowing for a reduction of acquisition time/injected dose in pediatric ^99m^Tc‐DMSA planar imaging.

## 1 INTRODUCTION

Renal cortical scintigraphy using ^99m^Tc‐dimercapto‐succinic acid (DMSA) is a well‐established method for detecting renal cortical defects, especially in pediatric patients with urinary tract infection (UTI) and vesicoureteral reflux (VUR).^1^, ^2^ In addition, ^99m^Tc‐DMSA scintigraphy is capable of quantifying renal uptake, which is of great clinical importance in the follow‐up and management of renal disease.^3^, ^4^

In clinical practice, pediatric ^99m^Tc‐DMSA scintigraphy suffers from motion artifacts. Although sedation can often be administered to restrain motion in pediatric patients, involuntary movements are bound to occur during sleep. Acquisition time reduction is likely to reduce the incidence of these motions; however, this method may also degrade the image quality and affect the diagnostic and quantitative performance of ^99m^Tc‐DMSA scintigraphy. One possible approach to improve image quality without increasing the administered dose is to use a conventional smoothing filter.^5^ In general, a smoothing filter can effectively reduce image noise; however, there is concomitant degradation in spatial resolution and a reduction in edge sharpness. In addition, in patients weighing less than 20 kg, a longer acquisition time is desirable to prevent image quality degradation in ^99m^Tc‐DMSA planar imaging.^6^ If the image quality improvement of short‐acquisition‐time images can be achieved, prolonged acquisition time would be unnecessary in such cases.

Recently, deep learning algorithms have achieved outstanding performance in the field of nuclear medicine.^7^ For instance, many recent studies have demonstrated the potential of converting ultra‐fast/low‐dose PET to full‐count PET using denoising tasks.^8^, ^9^, ^10^, ^11^, ^12^, ^13^, ^14^ Furthermore, the feasibility of reducing SPECT acquisition time has been investigated in myocardial perfusion imaging,^15^ bone imaging,^16^ and renal imaging.^17^ Lin et al. demonstrated that a trained three‐dimensional residual U‐Net model could generate full‐acquisition‐time reconstructed SPECT images from half‐acquisition‐time images, thereby reducing the acquisition time of ^99m^Tc‐DMSA SPECT imaging while maintaining diagnostic accuracy.^17^ However, to the best of our knowledge, no previous work has focused on fast scanning ^99m^Tc‐DMSA planar imaging.

Several works have already demonstrated that deep learning processing can achieve highly accurate noise rejection compared to conventional smoothing filter processing.^18^, ^19^ Tsuchiya et al. demonstrated that deep learning‐based images respected the tissue boundaries well and significantly reduced noise without losing quantitative information on PET images compared to images using a conventional Gaussian filter.^18^ Ito et al. reported that deep denoising super‐resolution convolutional neural networks provided significantly improved image quality compared to Gaussian processing in a low‐count acquisition of bone scintigraphy.^19^ Motivated by the success of deep learning in denoising tasks, the present study predicted full‐acquisition‐time images from short‐acquisition‐time ^99m^Tc‐DMSA planar images (1/5th of the regular acquisition time) using three well‐established architectures, namely DnCNN,^20^ Win5RB,^21^ and ResUnet.^22^ The purpose of this work was to evaluate the performance of predicted full‐acquisition‐time images from short‐acquisition‐time pediatric ^99m^Tc‐DMSA planar images using deep learning in terms of image quality and quantitative renal uptake measurement accuracy.

## 2 METHODS

### 2.1 Patients

Our institutional review board approved this study and waived the requirement for individual informed consent due to the retrospective nature of the study. We included consecutive pediatric patients under the age of 15 years who underwent ^99m^Tc‐DMSA planar imaging between April 2017 and March 2022. Twenty cases were excluded due to different scan sequences in which planar images were not acquired as dynamic data, while one case was excluded because the procedure was terminated in the middle of the examination as a result of arousal. Together, these criteria resulted in the inclusion of 155 cases, and the constructed datasets were divided into three separate subsets based on acquisition date: training (*n* = 109), validation (*n* = 23), and test (*n* = 23). The training set was used to train the model and make it learn the hidden features in the data. The validation set was used to provide an unbiased evaluation of the model fitted to the training set while tuning the model's hyperparameters. The test set was used to evaluate the performance of the predicted full‐acquisition‐time images from the final model in terms of image quality and quantitative renal uptake measurement accuracy.

Patient demographics are shown in Table 1. No significant differences in sex, age, height, weight, and injected activity were observed between the training, validation, and test data subsets (*p* > 0.05 for all). The patient's main diseases were VUR and/or UTI (*n* = 133), hydronephrosis (*n* = 17), and polycystic kidney (*n* = 5).

**TABLE 1:** Demographics of patients included in this study.

Parameter	Training	Validation	Test	*p*‐value
Number	109	23	23	–
Male/female	77/32	16/7	16/7	0.991
Age, median (IQR) years	1.7 (1.0−5.6)	1.2 (1.0−4.6)	2.0 (0.9−5.6)	0.697
Height, median (IQR) cm	79.1 (72.3−107.5)	76.4 (70.5−102.7)	80.0 (73.1−109.5)	0.682
Weight, median (IQR) kg	10.3 (8.9−17.4)	10.8 (8.4−17.1)	10.3 (9.5−17.1)	0.776
Injected activity, median (IQR) MBq	58.8 (49.4−75.7)	56.5 (47.7−67.7)	56.8 (54.7−71.4)	0.401

*Notes*: The Kruskal–Wallis test was used to calculate the *p*‐value for continuous variables. The chi‐square test was used for categorical variables.

Abbreviation: IQR, interquartile range.

### 2.2 ^99m^Tc‐DMSA planar imaging

^99m^Tc‐DMSA planar imaging was performed on a Symbia T16 SPECT/CT scanner equipped with a low‐energy high‐resolution collimator (Siemens Healthcare, Erlangen, Germany). The administered dose was determined according to the consensus guidelines for pediatric nuclear medicine published by the Japanese Society of Nuclear Medicine (JSNM).^23^ After 2 h of injection of the ^99m^Tc‐DMSA imaging agent, posterior planar images were acquired as dynamic data for 10 min at 1 min per frame. Composite images of ten frames were routinely used to detect renal cortical defects and quantify renal uptake. The matrix size was 256  ×  256, and the zoom factor was 1.78. The pixel size was 1.35 × 1.35 mm^2^. All images were downloaded from our Picture Archiving and Communication System (PACS) in a Digital Imaging and Communications in Medicine (DICOM) format at a 16‐bit grayscale.

### 2.3 Dataset preparation

**FIGURE 1:**
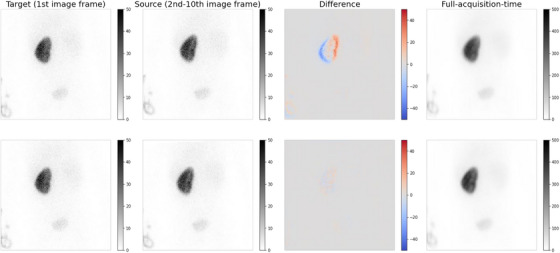
Example of frame registration of ^99m^Tc‐DMSA planar images (top: before registration, bottom: after registration). The left images are the target images (the first image frames), and the middle left images are the source images at a given frame. Of the ten frames, the 4th image frames are shown as representative images. The middle right images are the difference between the target and source images. Registration was performed to minimize the mean squared error between the two images. Misalignments are visible as red or blue shadows. When the two images are exactly aligned, the kidney will be gray on the differences. The right images are the corresponding full‐acquisition‐time images. Tissue boundaries in the full‐acquisition‐time images were well visualized by image registration.

According to the consensus guidelines for pediatric nuclear medicine published by the JSNM,^23^ the use of motion correction programs may be considered when the patient's body motion cannot be properly restrained. As reported by Polycarpou et al.,^24^ respiratory motion correction using an image‐based procedure is effective in achieving obtaining more uniform uptake and reducing motion blur. Therefore, image‐based motion correction was applied to the dynamic data to minimize the effect of motion artifacts during image acquisition and to generate high‐quality training data. In this procedure, each image frame was registered to the first image frame (Figure 1). Registration was performed using the Autograd Image Registration Laboratory (AIRLab) with a rigid transformation model in which the source image was rotated, translated, and scaled.^25^ Linear interpolation was used to apply the transformation to the source image. The registration was optimized over 100 iterations using mean squared error (MSE) as the similarity measure. The details of the AIRLab framework can be found on GitHub (https://github.com/airlab‐unibas/airlab).

We used images with 1/5th acquisition time (2 min) as the input data for deep learning because we could prepare the increased number of training images compared to the case using images with 1/10th acquisition time (1 min) by the following procedure. The short‐acquisition‐time (2 min) input data were obtained by adding two frames selected from a total of 10 frames. In this process, all possible pairs were used to increase the number of training images. In short, 45 input image subsets were obtained for each case, and 4 905 training image subsets, 1 035 validation image subsets, and 1 035 test image subsets were generated. The target data were the corresponding full‐acquisition‐time (10 min) image subsets.

### 2.4 Deep learning algorithms

**FIGURE 2:**
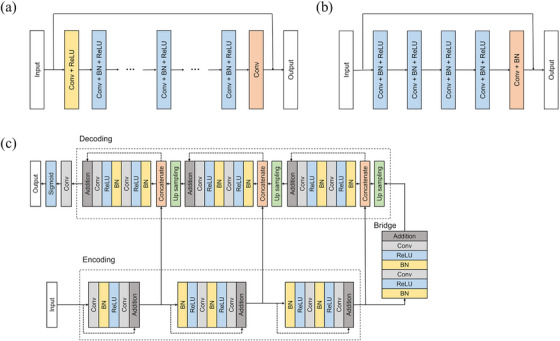
Model architectures, namely (a) DnCNN, (b) Win5RB, and (c) ResUnet, evaluated in this study. Conv represents convolution, BN represents batch normalization, and ReLU represents a rectified linear unit.

While various state‐of‐the‐art deep‐learning architectures have been proposed, we have used three well‐known deep learning algorithms, namely DnCNN,^20^ Win5RB,^21^ and ResUnet.^22^ The reasons for using these networks are twofold. First, these networks are based on residual networks, assuming that residual learning can facilitate training and address the degradation problem that occurs in deep neural network models. Second, these networks have shown promising results in generating pseudo images from input medical images in several works.^26^, ^27^ Figure 2 shows the model architecture used in this study. We implemented the models by using an open‐source deep learning library (PyTorch Lightning; https://www.pytorchlightning.ai/).

DnCNN has the basic unit including a 3 × 3 convolution (Conv) layer with 64 filters, a batch normalization (BN) layer, and a rectified linear units (ReLU) activation layer. The first layer includes a Conv layer and a ReLU layer, and the basic units are sequentially connected 15 times, followed by a 3 × 3 Conv layer for the last layer. The output image is obtained by subtracting the input image from the residual image.

Win5RB has two types of layers: (1) Conv+BN+ReLU, and (2) Conv+BN. The first type of layer is sequentially connected four times, followed by the second type of layer for the last layer. In addition, a shortcut skip connecting the input with the output image is added. A kernel size of 7 × 7 with 64 filters was used in this study.

ResUnet combines the strengths of both U‐Net and residual neural networks. The network consists of three parts: encoding, bridge, and decoding. The first part, also called down‐sampling, extracts the features of the data and reduces the size of the data. The last part, also called up‐sampling, expands and restores the data to the size of the original data. The middle part serves as a bridge connecting the encoding and decoding paths. All three parts are built with residual units consisting of two 3 × 3 convolution blocks and a skip connection operation. Each convolution block includes a BN layer, a ReLU layer, and a Conv layer.

Data standardization to achieve a zero mean of observed values and a standard deviation of 1 was performed before inputting images into the models. The loss function used was MSE, and the Adam optimizer^28^ was used to adjust model weights. The initial learning rate was set to 0.0001. The maximum number of training epochs was 50, and the batch size was 8. The model weight was saved when the validation loss was minimized. Training, validation, and testing of the model were performed in the following environment: CPU, IntelCore i7_9700F CPU at 3.00 GHz; GPU, NVIDIA GeForce RTX 2070 Super 8 GB; Framework, PyTorch Lightning 1.5.9; Language, Python 3.8.12, CUDA, 11.3; and CUDNN, 8.2.0. In our environment, the average implementation time from inputting images into the models to providing the post‐processed images was 0.39 ± 0.03 s, 0.21 ± 0.03 s, and 0.24 ± 0.03 s for DnCNN, Win5RB, and ResUnet, respectively,

### 2.5 Assessment of image quality

The accuracy of predicting full‐time images from short‐time images in the test sets was evaluated using three quantitative metrics, including the normalized mean squared error (NMSE),^29^ the peak signal‐to‐noise ratio (PSNR),^30^ and the structural similarity index metrics (SSIM)^31^ (Equations (1), (2), (3)). Moreover, these metrics were also calculated for short‐time images to provide insight into the noise levels and significant signal loss.

(1) $$\begin{array}{c} NMSE\left(x,\;y\right)=\frac{\sum {\left(y-x\right)}^{2}}{\sum y^{2}}\times \;100\;\left[\%\right] \end{array}$$

where *y* is the reference full‐time image, and *x* is the short‐time or predicted image.

(2) $$\begin{array}{c} PSNR\left(x,y\right)=20\times \log_{10}\left(\frac{MAX\left(y\right)}{\sqrt{MSE\left(x,y\right)}}\right)\;\left[dB\right] \end{array}$$

where *MAX* (*y*) indicates the maximum pixel value of the full‐time image, and MSE is the mean square error.

(3) $$SSIM\left(x,\;y\right)=\;\frac{\left(2\mu_{x}\mu_{y}+c_{1}\right)\left(2\sigma_{xy}+c_{2}\right)}{\left(\mu_{x}^{2}+\mu_{y}^{2}+c_{1}\right)\left(\sigma_{x}^{2}+\sigma_{y}^{2}+c_{2}\right)}\;$$

where *μ_x_* and *μ_y_* are the mean values of *x* and *y*, respectively. Furthermore, *σ_x_* and *σ_y_* represent the variances of *x* and *y*, respectively, and *σ_xy_* represents the covariance of *x* and *y*. Finally, *c*_1_ and *c*_2_ are regularization constants, defined as *c*_1_ = (0.01 × *L*)^2^ and *c*_2_ = (0.03 × *L*)^2^, where *L* is the dynamic range of intensity.

### 2.6 Accuracy of renal uptake

Quantitative parameters were calculated for the reference full‐time images, short‐time images, and three predicted full‐time images using the DRIP (Fujifilm Toyama Chemical Co., Ltd., Tokyo, Japan) software. Region of interests (ROIs) were placed on each kidney and background tissue of the reference full‐time images, and the ROIs were subsequently copied to other images.

Renal uptake of ^99m^Tc‐DMSA was calculated using the following equation:

(4) $$Renal\;uptake=\frac{U-B}{D}\times \frac{1}{k}\times 100\;\left[\%\right]$$

where *U* and *B* indicate kidney count and kidney background count, respectively, *D* is the total dose count, and k is a coefficient of kidney depth. Kidney depth was estimated from the equation previously reported by Tauxe et al.^32^ as follows:

(5) $$\begin{array}{c} Y=\left(0.82\;\times W-0.36\;\times H-0.06\;\times A+61.088\right)/10\;\left[cm\right] \end{array}\;\;$$

where *W*, *H*, and *A* represent the weight (kg), height (cm), and age (year), respectively.

### 2.7 Statistical analysis

Statistical analysis was performed with EZR,^33^ which is a modified version of R commander designed to add statistical functions frequently used in biostatistics. The significant level was set at *p* < 0.05.

Patient demographics were expressed as median (interquartile range) because the data were not normally distributed according to the Shapiro–Wilk test. Patient demographics were compared between the training, validation, and test data subsets using the Kruskal–Wallis test for continuous variables. The chi‐square test was used for categorical variables.

The Friedman test and subsequently post hoc analyses with the Wilcoxon signed rank sum test were used to compare NMSE, PSNR, and SSIM between short‐time, DnCNN, Win5RB, and ResUnet because these metrics were not normally distributed according to the Shapiro–Wilk test. Post hoc pairwise comparisons were adjusted for multiple comparisons using Bonferroni correction. Moreover, the voxel‐wise joint histograms with Pearson correlation were computed to explore the correlation between short‐time or predicted full‐time images with respect to the reference full‐time images.

The average and maximum counts, and renal uptake on each kidney were compared between reference full‐time, short‐time, and predicted full‐time images using the Friedman test and subsequently post hoc analyses with the Wilcoxon signed rank sum test. Post hoc pairwise comparisons were adjusted for multiple comparisons using Bonferroni correction. The difference in renal uptake compared to the reference full‐time images was evaluated using scatter plots with Pearson correlation and Bland–Altman plots.

## 3 RESULTS

Note that the results presented below are based on the test sets only.

### 3.1 Assessment of image quality

**FIGURE 3:**
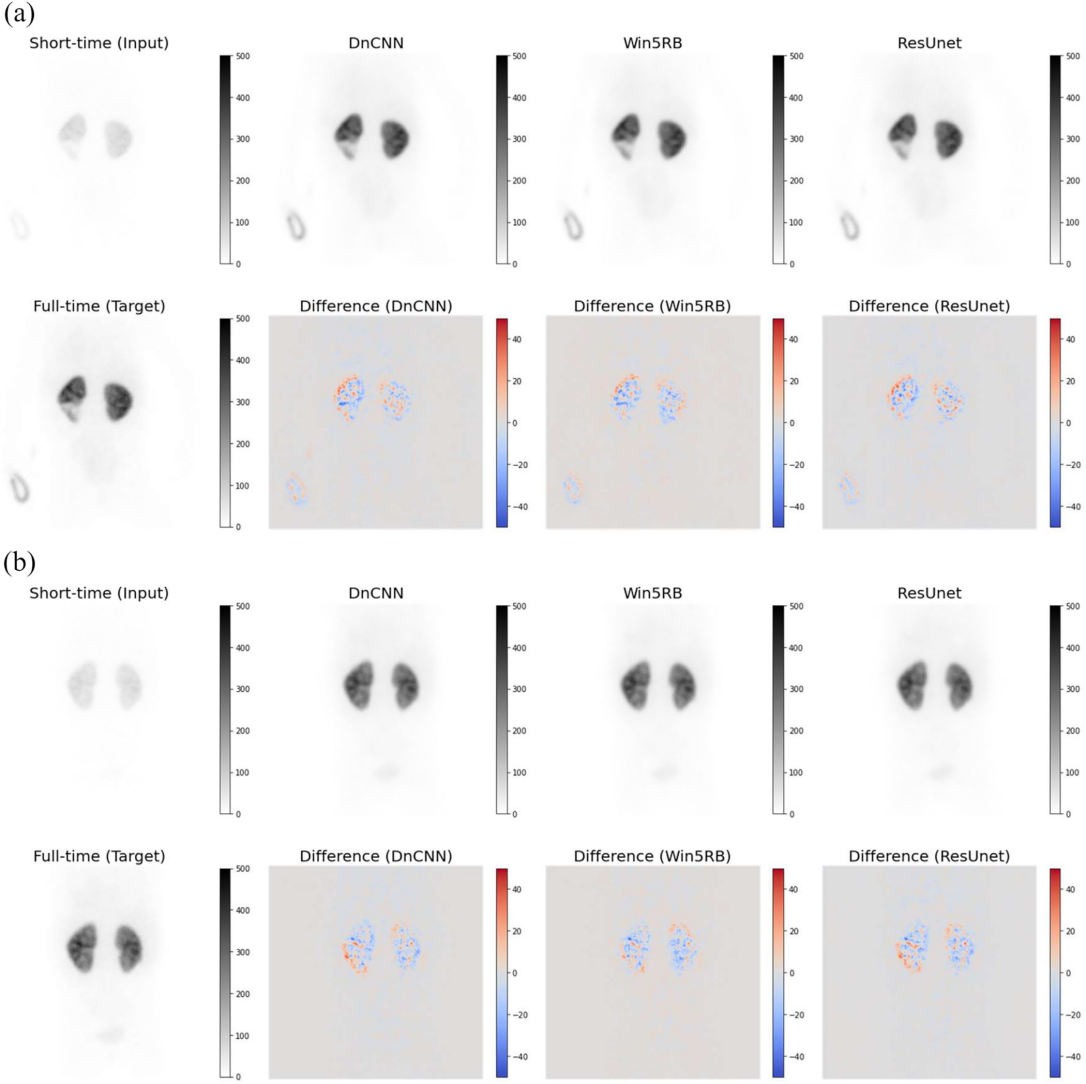
Representative ^99m^Tc‐DMSA planar images of (a) a 2‐year‐old male patient and (b) a 3‐year‐old female patient. The predicted full‐time images obtained by three deep learning models (DnCNN, Win5RB, and ResUnet) provide an almost similar visual appearance to the reference full‐time image. The colored images show the difference from the reference full‐time image. When the counts in the reference full‐time image are higher, the pixels are visible as red shadows, while when the counts in the predicted full‐time images are higher, the pixels are visible as blue shadows.

Table 2 summarizes the results of the image quality metrics, including NMSE (%), PSNR (dB), and SSIM. An objective improvement in image quality is reflected by a lower value of NMSE or a higher value of PSNR or SSIM. The predicted full‐time images from the three deep learning models (DnCNN, Win5RB, and ResUnet) showed a significant improvement in image quality compared to the short‐time images and a similar visual appearance to the reference full‐time images (Figure 3). All pairwise comparisons had *p*‐values < 0.001. In particular, the predicted full‐time images by ResUnet showed the lowest NMSE (median [interquartile range]: 0.4 [0.4−0.5] %) and the highest PSNR (55.4 [54.7−56.1] dB) and SSIM (0.997 [0.995−0.997]). In other words, the predicted full‐time images by ResUnet had significantly less noise and better image quality compared to those by the other models and the short‐time images.

**TABLE 2:** Image quality metrics of short‐time and predicted full‐time images relative to the reference full‐time images.

Parameter	Short‐time (2 min)	DnCNN	Win5RB	ResUnet
NMSE (%)				
Mean (SD)	64.5 (0.1)	1.7 (1.9)	1.5 (1.6)	0.5 (0.3)
Median (Q1, Q3)	64.5 (64.4, 64.6)	0.9 (0.6, 1.6)	0.9 (0.5, 1.7)	0.4 (0.4, 0.5)
PSNR (dB)				
Mean (SD)	33.5 (1.2)	51.3 (3.9)	51.4 (3.9)	54.9 (2.0)
Median (Q1, Q3)	33.6 (32.6, 34.1)	52.2 (49.1, 54.4)	52.4 (48.5, 54.7)	55.4 (54.7, 56.1)
SSIM				
Mean (SD)	0.862 (0.016)	0.995 (0.002)	0.994 (0.004)	0.996 (0.002)
Median (Q1, Q3)	0.860 (0.854, 0.875)	0.996 (0.994, 0.997)	0.996 (0.992, 0.997)	0.997 (0.995, 0.997)

Abbreviations: NMSE, normalized mean square error; PSNR, peak signal‐to‐noise ratio; SSIM, structural similarity index; SD, standard deviation; Q1, the first quartile; Q3, the third quartile.

**FIGURE 4:**
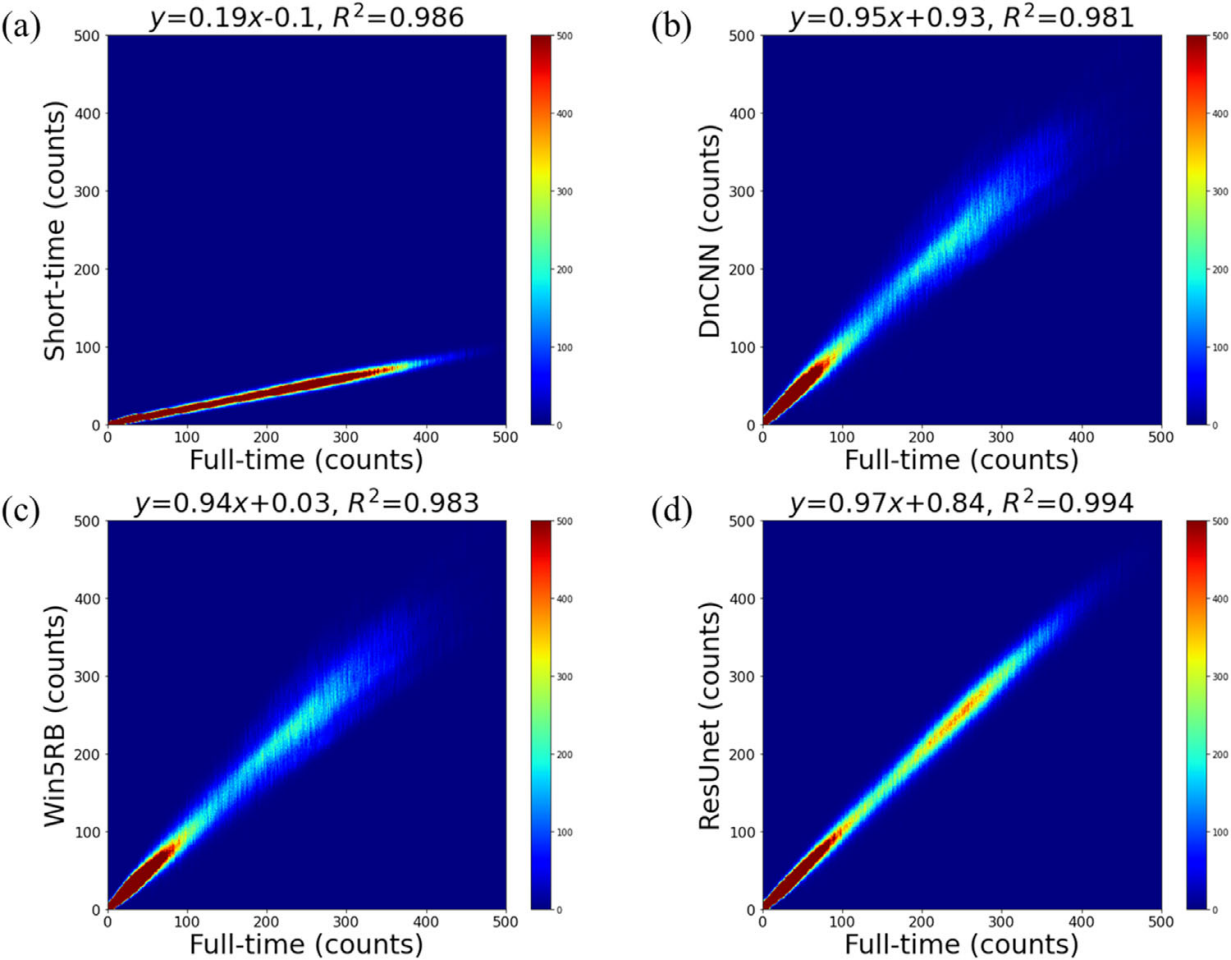
Joint voxel‐wise histogram analysis of (a) short‐time images and predicted full‐time images using (b) DnCNN, (c) Win5RB, and (d) ResUnet versus full‐acquisition‐time images.

Figure 4 shows the results of the voxel‐wise joint histogram analysis for the short‐time and predicted full‐time images. The short‐time images showed a strong correlation with the reference full‐time images (*R* ^2^ = 0.986, *p* < 0.001). The scatter plots showed a higher correlation between ResUnet and the reference full‐time images (*R* ^2^ = 0.994, *p* < 0.001) compared to DnCNN (*R* ^2^ = 0.981, *p* < 0.001) and Win5RB (*R* ^2^ = 0.983, *p* < 0.001). Of all the images, ResUnet had the highest correlation with the reference full‐time images.

### 3.2 Accuracy of renal uptake

The median ROI size was 28.4 (24.9−37.8) mm^2^ on each kidney and 8.8 (7.5−9.3) mm^2^ on background tissue. Table 3 shows the average and maximum counts, and renal uptake on each kidney for the reference full‐time, short‐time, and predicted full‐time images. There were significant differences in the average and maximum counts of each kidney between the pairs of reference full‐time, short‐time, and three predicted full‐time images (*p* < 0.01), except for between DnCNN and ResUnet (*p* > 0.05). As for renal uptake, all pairwise comparisons had *p*‐values < 0.05.

**TABLE 3:** Comparison of the average and maximum counts, and renal uptake of kidney

Parameter	Full‐time (10 min)	Short‐time (2 min)	DnCNN	Win5RB	ResUnet
Average counts					
Mean (SD)	210.1 (29.5)	41.6 (5.9)	211.3 (29.2)	212.1 (29.8)	211.1 (29.6)
Median (Q1, Q3)	206.9 (189.8, 223.2)	41.1 (37.5, 44.5)	208.4 (191.1, 224.9)	209.4 (191.5, 226.3)	208.4 (190.5, 225.8)
Maximum counts					
Mean (SD)	392 (55)	88 (12)	380 (51)	384 (52)	380 (53)
Median (Q1, Q3)	389 (351, 430)	88 (80, 96)	381 (342, 413)	381 (341, 417)	376 (341, 411)
Renal uptake (%)					
Mean (SD)	21.3 (4.6)	21.1 (4.6)	21.4 (4.6)	21.5 (4.7)	21.4 (4.6)
Median (Q1, Q3)	21.1 (18.6, 23.1)	20.8 (18.4, 23.1)	21.2 (18.7, 23.4)	21.3 (18.8, 23.5)	21.1 (18.7, 23.4)

Abbreviations: SD, standard deviation; Q1, the first quartile; Q3, the third quartile.

**FIGURE 5:**
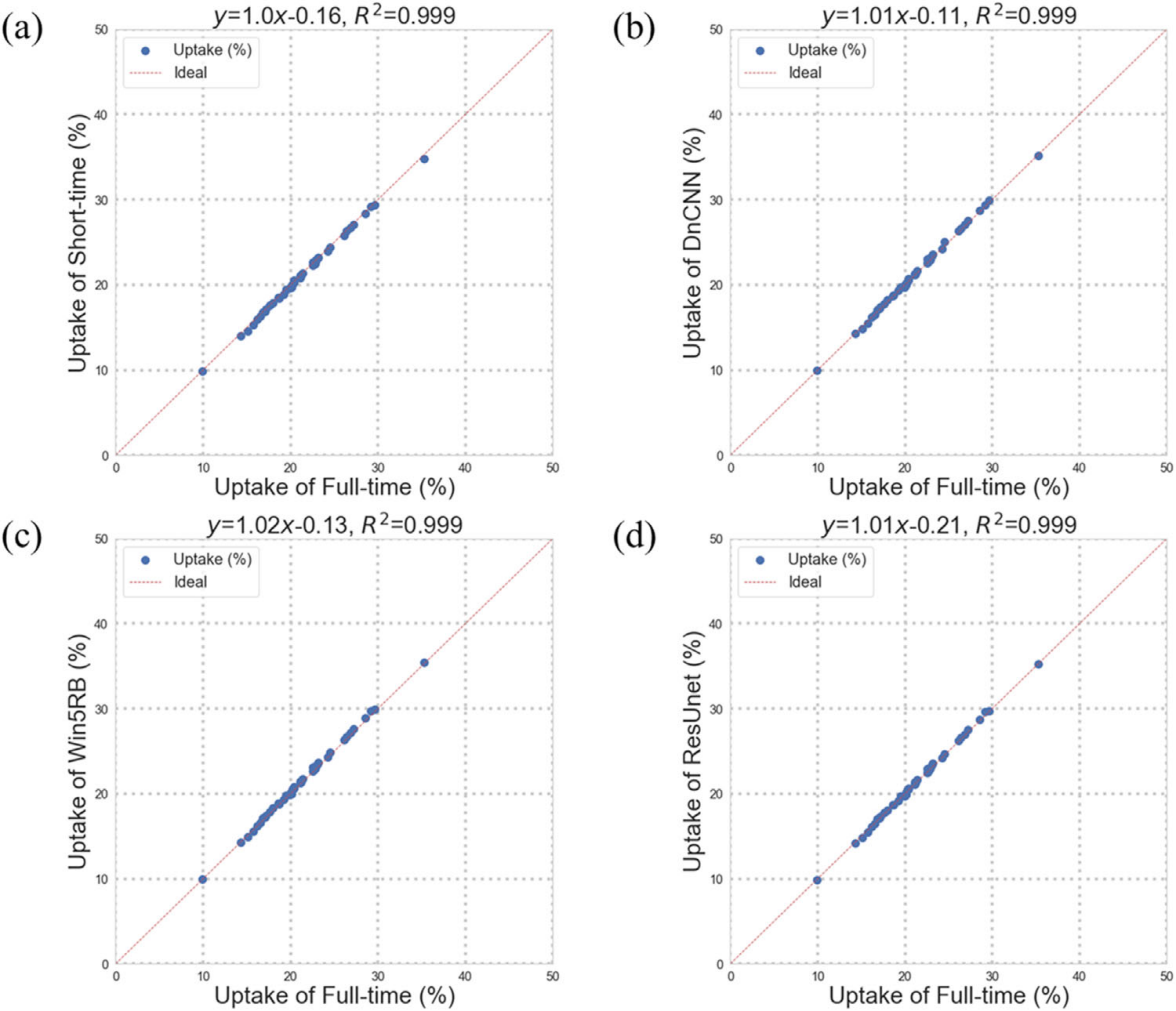
The scatter plots of renal uptake in (a) short‐time images and predicted full‐time images using (b) DnCNN, (c) Win5RB, and (d) ResUnet versus full‐acquisition‐time images.

**FIGURE 6:**
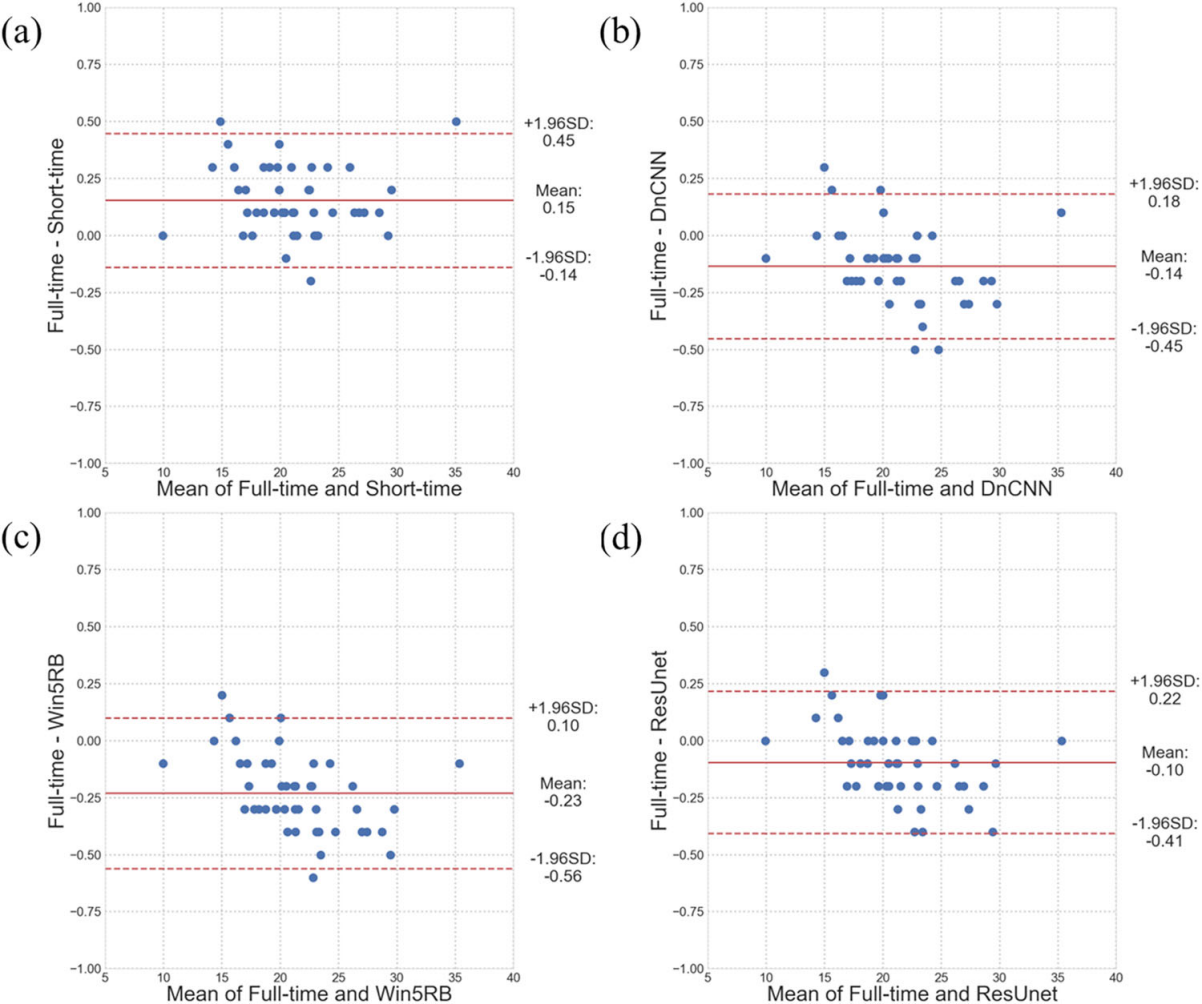
The Bland–Altman plots of renal uptake differences in (a) short‐time images and predicted full‐time images using (b) DnCNN, (c) Win5RB, and (d) ResUnet versus full‐acquisition‐time images. Solid and dashed lines denote the mean and 95% confidence interval (CI) of the renal uptake differences, respectively.

The scatter plots and Bland–Altman plots of renal uptake are shown in Figures 5 and 6, respectively. An extremely high correlation (*R* ^2^ > 0.999 for all) was obtained for all short‐time images and three predicted full‐time images. The Bland–Altman plots showed that the lowest bias (−0.10) of the renal uptake was in ResUnet. The lowest variance (95% confidence interval [CI]: −0.14, 0.45) of the renal uptake was obtained in the short‐time images. For predicted images, DnCNN (95% CI: −0.45, 0.18) and ResUnet (95% CI: −0.41, 0.22) showed lower variance compared to Win5RB (95% CI: −0.56, 0.10).

## 4 DISCUSSION

In this work, we evaluated the performance of predicted full‐acquisition‐time images from short‐time images with only 1/5th acquisition time in pediatric ^99m^Tc‐DMSA planar imaging using deep learning. We found that NMSE, PSNR, and SSIM were all significantly better for predicted full‐time images compared to short‐time ones. In particular, our results demonstrated the superior performance of ResUnet over DnCNN and Win5RB for predicting full‐acquisition‐time images in terms of image quality assessment. Furthermore, the difference in renal uptake for the predicted full‐time images was the smallest in ResUnet compared to the reference full‐time images. These results highlight that the ResUnet model is capable of producing images that are comparable to the original full‐acquisition‐time ones.

Image quality degradation was inevitable in short‐time images compared with full‐time images, which theoretically have the best image quality. According to Fujiwara et al.,^6^ a ^99m^Tc‐DMSA renal scan using the dose recommended by the JSNM consensus guidelines can be performed with a 3‐min acquisition time in patients weighing 20 kg or more. In contrast, for patients weighing less than 20 kg, a longer image acquisition time should be considered since increased noise exceeds the lower limit of diagnosable images with a 3‐min acquisition at the dose recommended by the JSNM consensus guidelines.^6^ The average body weight of our population was 14.3 ± 8.2 kg, thus an acquisition time of 2 min in the input data may not have been sufficient to obtain diagnosable images. Therefore, our deep learning‐based methods may be useful to improve the image quality of fast ^99m^Tc‐DMSA planar imaging.

Interestingly, our results showed that both short‐time (2 min) images and predicted full‐time images maintained the accuracy of renal uptake measurement. Based on the statistical analyses, there were still significant differences in all pairwise comparisons of renal uptake. However, the *p*‐values would be affected by the variability of the data. Therefore, we believe that these differences were clinically quite small. Actually, the Bland–Altman plots for the short‐time and the three predicted full‐time images showed only a small bias with a maximum of −0.23 and a small variance with a maximum of 0.66 (95% CI: −0.56, 0.10) in renal uptake compared to the reference full‐time images.

Motion during the acquisition process is bound to impair the visualization of renal cortical defects. Sedation may therefore be required particularly in younger children or uncooperative older children who must remain motionless for prolonged periods.^2^ Nevertheless, involuntary movements, such as respiratory movements, are bound to occur during sleep. According to Panandiker et al.,^34^ renal motion in pediatric patients younger than 9 years of age ranges from 5 to 25 mm in orientation‐specific directions on 4‐D computed tomography. Previous studies have successfully applied patient motion detection and correction methods in dynamic renal scintigraphy.^35^, ^36^ Another study demonstrated that respiratory motion correction using an image‐based procedure provided more uniform uptake and reduced motion blur in SPECT myocardial perfusion imaging.^24^ Therefore, to improve the image quality of our training data, we applied motion correction by rigid transformations in dataset preparation. This procedure well visualized the tissue boundaries in the full‐acquisition‐time image, as shown in Figure 1. By preparing these motionless training data, superior output images of deep learning would be achieved compared to the case of using the training data without motion correction. This also highlights the value of reducing the acquisition time of ^99m^Tc‐DMSA planar imaging to reduce the likelihood of motion artifacts.

The present study evaluated three well‐known deep learning algorithms (DnCNN, Win5RB, and ResUnet), and our findings showed that ResUnet outperformed the other models in terms of image quality assessment and quantitative renal uptake measurement. Such behavior could have been predicted because the combination of U‐Net and residual neural network in ResUnet facilitates information propagation without degradation.^22^ U‐Net‐based networks have been very successful in converting ultra‐fast/low‐dose PET to full‐count PET images,^8^, ^13^ ultra‐high speed SPECT bone imaging,^16^ and reducing the scan time of renal ^99m^Tc‐DMSA SPECT imaging.^17^ In contrast to previous works, the present study demonstrated that deep learning‐based approaches can generate full‐acquisition‐time pediatric ^99m^Tc‐ DMSA planar images from short‐acquisition‐time images and significantly reduce the acquisition time from 10 to 2 min. Since acquisition time and injected dose are the two primary parameters that affect image quality, deep learning‐based approaches that enable shorter acquisition times can effectively reduce the injected dose when the total acquisition time remains the same.

Our study had a few limitations. First, this study was a retrospective design using datasets from a single institution. Therefore, the generalization ability of these models to ^99m^Tc‐DMSA planar images obtained at external institutions using other manufacturers’ machines is unknown. In future work, it will be necessary to develop the algorithms using images acquired on multiple scanners. Second, the diagnostic accuracy of detecting cortical defects was not investigated. Instead, the predicted full‐time images were evaluated not only by traditional metrics but also by quantitative renal uptake. Finally, the feasibility of a much shorter acquisition time (e.g., 1 min) rather than 2 min could not be evaluated due to a lack of training datasets. Forty‐five input images could be obtained from 10 frames for each case when the acquisition time of input data was set to 2 min, while only 10 input images could be generated when the acquisition time was set to 1 min. For these reasons, the cutoff points for training data still need to be considered to determine the appropriate approach for data set construction and to ensure the accuracy and validity of the dataset. Further studies on a larger population are required to determine the lower limit of acquisition time reduction using deep learning.

## 5 CONCLUSION

In this work, we focused on generating images that are comparable to the original full‐acquisition‐time images from fast ^99m^Tc‐DMSA planar images with only 1/5th acquisition time. The comprehensive results showed that deep learning‐based methods, particularly ResUnet architecture, could significantly improve the image quality and maintain the accuracy of the renal uptake measurement. The proposed method was able to achieve a fivefold reduction in acquisition time for pediatric ^99m^Tc‐DMSA planar imaging, which can in turn can reduce the likelihood of motion artifacts in clinical settings. This method would also be useful to effectively reduce the injected dose when the total acquisition time remains the same.

## AUTHOR CONTRIBUTIONS

Shota Ichikawa carried out the data collection, algorithm construction, and the writing and editing of the article. Hiroyuki Sugimori performed supervision and reviewing and editing of the article. Koki Ichijiri analyzed the patient data regarding renal uptake. Takaaki Yoshimura contributed to the reviewing and editing of the article. Akio Nagaki contributed to the study design, project administration, and reviewing and editing of the article. All authors read and approved the final manuscript.

## CONFLICT OF INTEREST STATEMENT

The authors declare no conflicts of interest.

## Data Availability

The code generated during the current study is available from the corresponding author upon reasonable request. However, the image datasets presented in this study are not publicly available due to ethical reasons.
